# Online-Processing of Grammatical Gender in Noun-Phrase Decoding: An Eye-Tracking Study With Monolingual German 3rd and 4th Graders

**DOI:** 10.3389/fpsyg.2019.02586

**Published:** 2019-11-15

**Authors:** Jürgen Cholewa, Isabel Neitzel, Annika Bürsgens, Thomas Günther

**Affiliations:** ^1^Institute of Special Education, Heidelberg University of Education, Heidelberg, Germany; ^2^Study Programme Teaching and Research Logopedics (M.Sc.), Department of Neurology, Medical Faculty, RWTH Aachen University, Aachen, Germany; ^3^Child Neuropsychology Section, Department of Child and Adolescent Psychiatry, Psychotherapy and Psychosomatics, Medical Faculty, RWTH Aachen University, Aachen, Germany; ^4^Faculty of Health, Zuyd University, Heerlen, Netherlands

**Keywords:** grammatical gender, visual world paradigm, online auditory comprehension, online reading comprehension, psycholinguistic decoding

## Abstract

Like many other languages, German employs a linguistic category called “grammatical gender.” In gender-marking languages each noun is assigned to a particular gender-class (in German: masculine, feminine or neuter) and other words in a sentence which are grammatically controlled by the noun are marked by particular morphemes according to the noun’s gender feature – so called gender agreement. Within psycholinguistic theories of language comprehension, it is often assumed that gender agreement might help to predict the continuation of a sentence on grammatical grounds and to reduce the lexical search space for the next words emerging within the speech signal. Thus, gender agreement relations may provide a means to make the comprehension process more effective and targeted. The aim of the current study was to assess whether monolingual German 3rd and 4th grade primary school children make use of gender agreement in online auditory comprehension and whether different gender cues interact with each other and with semantic information. A language-picture matching task was conducted in which 32 children looked at two pictures while listening to a noun phrase. Due to features of the German gender system, the target picture corresponding with the noun phrase could be predicted shortly after stimulus onset on account of gender agreement relations. The predictive impact of grammatical gender agreement on noun-phrase decoding was investigated by measuring the time course of eye-movements onto the target and distractor pictures. The results confirm and extend previous findings that gender plays a role in predictive online comprehension of gender-marking languages like German, and that even primary school children are able to make use of this grammatical device.

## Introduction

Speed and effectiveness of information processing in spoken language comprehension has attracted much attention in psycholinguistic research and is emphasized as representing a remarkable ability in adults and typically developing children (e.g., Friederici, 2002; Treiman et al., 2003; Borovsky et al., 2013). Accordingly, it has been claimed that slowed and inefficient psycholinguistic processing can lead to comprehension deficits in children with developmental language impairment (Leonard et al., 2007).

Successful comprehension depends on the listeners’ ability to rapidly analyze and integrate linguistic information from different domains (e.g., phonological, semantic, morpho-syntactic), while the sentence unfolds with a rate of approximately three syllables per second (Montgomery, 2005). First, the comprehension process has to assort the incoming string of phonemes into substrings that correspond with phonological word forms (lexemes), then it has to access the semantic and grammatical features of the lexemes at the lemma-level of the mental lexicon and to parse the words into syntactic phrases (e.g., noun phrases and verbal phrases) (Cutler and Clifton, 1999; Levelt et al., 1999; Friederici and Weissenborn, 2007).

Moreover, in order to keep pace with the rapidly incoming and decaying acoustic signal listeners should predict probable continuations of a sentence or phrase as soon as possible and these probabilistic predictions have to be updated permanently as soon as new linguistic information reaches the decoding system (e.g., Federmeier, 2007; Jaeger and Snider, 2013; Pickering and Garrod, 2013).

For example, when decoding German noun phrases (e.g., *ein freundlicher Hund* ‘a friendly dog’) lexical features of the noun can be predicted on account of morpho-syntactic and semantic features of preceding articles and adjectives (*ein freundlicher*… ‘a friendly…’) as outlined in detail below.

In the example mentioned above listeners could predict that the noun is animate (e.g., an animal or a person) as soon as the adjective *freundlicher* ‘friendly’ is presented, since unanimated objects are probably not attributed as being *friendly* even though the semantic evidence is not unambiguous (e.g., *a friendly letter*) (cf. Aitchison, 1997). In an eye-tracking experiment, Borovsky et al. (2013) could show that listeners in fact updated and adjusted semantically based predictions in the course of an ongoing sentence. The authors presented simple transitive sentences (e.g., *the pirate*^AGENT^ *hides*^ACTION^ *the treasure*^THEME^) in a four-choice picture matching task. While the target picture depicted an object representing the theme (a treasure chest), two distractor pictures showed semantically related objects, one related to the agent (e.g., a pirate ship) and the other to the action (a bone). The third distractor picture was unrelated to either of the constituents (a cat). The target picture attracted about 75% of looks at the end of the sentence, indicating that the sentences were understood correctly. However, the probability of looks onto the agent-related distractor picture increased immediately after the first noun phrase was presented (the agent) and shifted more often to the action-related distractor (the bone) after the verb was spoken. The unrelated distractor remained widely unnoticed from the onset to the end of the sentence. Thus, while listening to the sentences, the participants took into consideration various semantically plausible continuations and the eye-movement patterns revealed an online adaptation of these predictions due to the semantic information provided successively within the sentences.

Another source of predictability in the example given above (*ein freundlicher*… ‘a friendly…’) is the grammatical gender of the article and the adjective, which is masculine. In German, gender is an inherent grammatical feature of all nouns accessible from the mental lexicon and transferred to other words in the syntactic domain of the noun according to gender agreement rules (Corbett, 1991). Linguistically, the German gender system helps to establish coherence between words belonging together within a noun phrase (van Berkum, 1996; Weber, 2001; Menzel, 2004). Hence, gender is an example par excellence for a linguistic category suitable to predict continuations in sentence comprehension.

According to the grammatical rules of gender agreement in German, a highly probable lexical successor of *ein netter* ‘a nice…’ would be a noun from the masculine gender class (e.g., *Hund*^MASC^′ ‘dog’), since the article *ein*^MASC^ and the adjective *netter*^MASC^ are both marked as masculine and must agree with the following noun they are headed by. A noun from one of the other two gender classes in German, namely feminine (e.g., *Katze*^FEM^ ‘cat’) or neuter (e.g., *Schaf*^*NEU*^ ‘sheep’) cannot be used in this grammatical context, since this would lead to an ungrammatical structure, violating the gender agreement rules.

According to Friederici and Jacobsen (1999), beneficial effects of gender agreement on lexical access could be explained in the following way. Whenever a noun phrase consisting of an article and a noun has to be decoded, the incoming gender marking provided by the article leads to an activation of a gender node which connects all words from the same gender category. From here, the activation spreads out to all agreeing nouns and this, in turn, can lead to a faster lexical access of the noun (cf. Neumann, 2001). Alternatively, predicting features of upcoming words can be considered beneficial in terms of processing efficiency. Since only a smaller lexical cohort needs to be checked against the phonological input, only partial phonological information (e.g., the first sound of the noun) may be sufficient to rule out phonological competitors of the target noun (Brouwer et al., 2017).

Further accounts to explain the effects of gender agreement, e.g., in terms of sound-based contingencies between the noun and its forerunner word have been proposed by Dahan et al. (2000). Since gender agreement restricts noun-phrase structures in many other languages as well, (e.g., Dutch, Hebrew, Spanish, and French), its psycholinguistic role attracted much attention. In a seminal paper, Dahan et al. (2000) conducted an eye-tracking experiment with French adult listeners in order to explore whether gender information indicated by the form of a definite article (*le* ‘the^MASC^’ or *la* ‘the^FEM^’) facilitates access to a succeeding noun (e.g., *bouton*^MASC^ ‘button’) and inhibits activation of a phonological similar competitor from a different gender category (e.g., *bouteille*^FEM^ ‘bottle’). In French, each noun is assigned to one of two gender classes and gender is indicated only by the singular forms of the definite article, while the plural form (*les* ‘the^PLUR^’) is neutralized with respect to gender. In the experiment, the French participants were presented with a four-choice noun phrase-picture matching task, in which the target (e.g., *bouton*^MASC^), a phonological neighbor from a different gender category (e.g., *bouteille*^FEM^) and two distractor pictures without phonological similarity to the target and from a different gender class were presented together with a sentence. The participants were asked to click onto the picture that matches the sentence (e.g., *cliquez sur le bouton* ‘click on the^MASC^ button^MASC^’). In a second experimental condition, the number of the depicted objects was changed (plural instead of singular) and consequently the instructions contained the (gender-neutralized) plural form of the definite article (e.g., *cliquez sur les boutons* ‘click on the^MASC/FEM^ buttons^MASC^’). In both conditions, eye movements onto the target and distractor pictures were measured in order to assess effects of gender information onto lexical access. Interestingly, within the (gender-neutralized) plural condition, the picture of the phonological competitor attracted significantly more attention than the two unrelated distractor pictures, while this effect completely disappeared within the singular condition. The authors assumed that the gender information provided by the article in the singular condition might have inhibited the activation of all nouns from other gender categories even if these nouns actually belonged to the same phonological cohort as the target noun.

Furthermore, in several studies from different gender marking-languages online processing of gender information in noun phrase decoding was explored using an eye-tracking-approach, originally introduced by Johnson (2005). Johnson prompted Dutch toddlers (26–30 months) to look at two pictures while listening to noun phrases consisting of an article and a noun (e.g., *het*^NEU^ *boek*^NEU^ ‘the book’). The noun phrases were divided into two experimental conditions. In the so-called informative trials, the target picture (here a book) was presented next to a distractor picture representing a noun from a different gender class (e.g., *bal*^Com^ ‘ball’). In the so-called uninformative trials, both pictures were selected from the same gender category. It was shown that the toddlers’ eyes shifted to the target pictures more quickly whenever they listened to noun phrases beginning with articles that provide informative gender marking.

Adopting this paradigm and the informative/uninformative distinction, Lew-Williams and Fernald (2007) conducted a study with Spanish adults and toddlers (aged 2;10 to 3;6 [years;months]). In Spanish, nouns, articles and adjectives agree with respect to their gender category (masculine or feminine). As in Johnson’s study, the depicted nouns were either from different gender categories (informative: *la pelota*^FEM^ ‘the ball’ and *el zapato*^MASC^ ‘the shoe’) or from the same gender class (uninformative: e.g., *la pelota*^FEM^ ‘the ball’ and *la galleta*^FEM^ ‘the cookie’). Both adults and toddlers identified the target pictures more quickly in the informative trials (gender processing). However, the time needed to shift to the target was longer in case of the toddlers, indicating that the children required more time to process gender agreement information (processing speed). Thus, development and maturation of an efficient gender processing system seems to be not only a matter of linguistic competence but of processing speed as well.

Melançon and Shi (2013) reported similar observations from an eye-tracking study with 30-months-old French infants. In French, too, nouns, articles and adjectives agree with respect to their gender category (masculine or feminine). The participants in this experiment listened to noun phrases consisting of an article, an adjective and a noun (e.g., *la^FEM^ mignonne^FEM^ girafe^FEM^* ‘the pretty giraffe’, *la^FEM^ mignonne^FEM^ grenouille^FEM^* ‘the pretty frog,’ *le^MASC^ mignon^MASC^ soulier^MASC^* ‘the pretty shoe’. While listening to the auditory input, the infants were presented two pictures representing either two objects from different gender categories (*giraffe* and *shoe*, informative) or from the same gender class (*giraffe* and *frog*, uninformative). The adjectives were introduced in order to provide more time for the gender information to unfold their predictive effects. Fixation-time onto the target and the distractor picture were analyzed with a particular focus on the time-unit between the onset of the noun phrase and the onset of the noun. In the informative trials, the recognition of the target picture was achieved before the noun was heard while in the uninformative trials, target recognition was delayed, as infants waited until they heard the noun form in order to select the target. This eye-movement pattern clearly indicates that the French children relied on gender information to anticipate the target, if possible.

In a recent study, Brouwer et al. (2017) used a similar task to investigate whether the eye-movement patterns of Dutch children between 4 and 7 years and of adults were influenced by gender agreement information in online language comprehension. As within the French study reported above, the participants listened to noun phrases consisting of an article, an adjective and a noun. Again, visual displays were provided with two objects, depicting nouns of either the same gender category (uninformative) or from different gender classes (informative). The authors formed the hypothesis that preferential gazes on the target in case of informative trials could potentially differ from those in the uninformative trials in two psycholinguistically distinct ways, namely there could be a predictive or facilitative use of gender information. Predictive gazes, defined as those prior to the onset of the noun, were assumed to reflect predictions on purely morpho-syntactic grounds (i.e., gender agreement) without relying on information concerning the phonological form of the noun itself. In contrast, a facilitative use of gender information was assumed to be indicated by preferential gazes on the target after the first phonemes of the noun were presented.

Results from the study of Brower and colleagues revealed a predictive use of gender information for the adult participants. However, the eye-movement patterns observed for the children differed with respect to their use of gender information. While those that were assumed to be less proficient due to their scores in a gender production task showed facilitatory gazes, the proficient children showed the same eye-movement pattern as observed for the adults. Thus, maturation of an efficient gender processing system might also be a matter of shifting from facilitatory to predictive processing strategies.

First evidence for the use of gender agreement in German noun phrases came from three eye-tracking experiments conducted by Hopp (2013), Hopp and Lemmerth (2016), and Lemmerth and Hopp (2017). Even though the main focus of these studies was on gender processing in L2-learners of German, control data with native speakers were collected (Hopp, 2013: *N* = 20; *M* = 21;0 [years;months], *SD* = 3;1, Hopp and Lemmerth, 2016: *N* = 15; *M* = 27;4 [years;months], *SD* = 6;1, Lemmerth and Hopp, 2017: *N* = 15; *M* = 8;0 [years;months], *SD* = 1;1. In all three experiments the contrast between informative and uninformative trials was essential.

In Hopp’s experiment, the participants looked at a set of same-colored drawings depicting objects (e.g., a yellow skirt, a yellow button, a yellow playing card) while listening to a sentence containing a noun phrase which consisted of a definite article, an adjective and a noun (e.g., *wo ist der^MASC^ gelbe*^MASC^ *Knopf*^MASC^ ‘where is the yellow button’). In the informative trials the two distractor objects were from another gender class than the target object, while in case of the uninformative trials all objects belonged to the same gender class. The author expected that the time needed to shift from the distractor pictures to the target (after the determiner was spoken) should be shorter in the informative trials. However, the predictively relevant gender information in Hopp (2013) was provided by the definite articles only (e.g., *der*^MASC^ *gelbe*^MASC^ *Knopf*^MASC^ ‘the yellow button,’ *die*^FEM^ *gelbe*^FEM^ *Karte*^FEM^ ‘the yellow card,’ *das*^*NEU*^ *gelbe*^*NEU*^ *Kleid*^*NEU*^ ‘the yellow dress’). Due to the specificities of the German gender system, adjectives following a definite article are homophonic and consequently ambiguous with respect to gender class (*gelbe*^MASC/FEM/NEU^ ‘yellow’). Thus, the adjectives did not provide predictively valuable information. There was merely more time for the gender information at the article to unfold its predictive impact.

The items presented in Hopp and Lemmerth (2016) were basically the same as those in Hopp (2013) despite one essential modification in one of the experimental conditions: Instead of definite articles, the noun phrases began with indefinite articles – either from the masculine or from the neuter gender class. As a consequence, the gender feature of the article did not allow to predict the target picture of informative trials, because indefinite articles that belong to the masculine and neuter gender class are homophonic in German (e.g., *ein^MASC^ gelb-er^MASC^ Käse^MASC^* ‘a yellow cheese’; *ein^*NEU*^ gelb-es^*NEU*^ Kleid*^*NEU*^ ‘a yellow dress’). However, the suffix attached to the adjectives unambiguously allowed to recognize its gender class (-*er*^MASC^, -*es*^*NEU*^) and hence – due to gender agreement - of the target noun. Thus, in Hopp and Lemmerth (2016) the predictively valuable gender information was provided only by the adjective immediately preceding the noun (in contrast to Hopp, 2013). Consequently, the time available for a predictive use of the gender information was shorter.

Additionally, in both experiments (Hopp, 2013; Hopp and Lemmerth, 2016) the researchers controlled the influence of possible predictively valuable semantic information provided by the meaning of the adjectives. For that purpose, only one of three color adjectives (yellow, red or green) or one of two size-adjectives (big and small) was used. The color adjectives were assumed to be semantically uninformative with respect to noun prediction because the objects were not depicted in a naturalistic way, but the objects were same-colored. As far as size-adjectives were concerned, they combined with the nouns in a way that the semantic features could not allow the prediction of the noun on semantics as well.

In both experiments eye-movements revealed that the native adult participants used gender agreement information provided by either the definite article or the gender-indicating adjectives in order to select the target picture. A significant preference to look at the target picture in the informative trials was observed immediately after presentation of the article in Hopp (2013) and immediately after presentation of the adjective in Hopp and Lemmerth (2016). However, even in the informative trials a considerable amount of uncertainty remained after gender information could be processed, since the proportion of looks onto the target did not exceed 50% in Hopp (2013) and 40% in Hopp and Lemmerth (2016). Thus, the participants’ gender-based predictions were probabilistic in nature and alternative continuations were still taken into account until the nouns’ word form became at least partially available.

In a recent study, Lemmerth and Hopp (2017) combined similar designs as used in Hopp (2013) and Hopp and Lemmerth (2016) in order to investigate online gender processing in 7–9 years old German speaking children. As in the two preceding studies the main focus was on bilingual language processing (Russian–German). However, with respect to our study the results obtained for the monolingual control group are of particular relevance, since we aim to investigate gender processing in almost the same age group. As in the two earlier studies, Lemmerth and Hopp (2017) examined processing of gender information provided by articles and adjectives separately. For that purpose, the language stimuli presented to the children for noun-phrase–picture matching, were assigned to one of two linguistically defined conditions. In both conditions the children were asked to look at a target picture that corresponded with a noun phrase. In condition 1 only the definite article allowed to predict the target picture unambiguously (e.g*., Wo ist der blaue Eimer?* ‘where is the blue bucket?’; distractor picture e.g., *eine blaue Bank* ‘a blue bench’) and in condition 2 only the adjective (e.g., *Wo ist ein kleiner gelber Eimer?* ‘where is a small yellow bucket?’; distractor picture e.g., *ein kleines gelbes Kissen* ‘a small yellow cushion’).

As in the preceding studies, only in half of the items of both conditions, the distractor pictures could be ruled out due to gender information. In the other half of the items in both conditions, the target picture could only be identified by recognizing the noun itself, since target and distractor pictures were selected from the same gender category.

As a dependent variable Lemmerth and Hopp determined the first look at the target picture after onset of the definite article and onset of the noun (condition 1) or offset of the adjective and onset of the noun (condition 2). The children showed shorter reaction times for items with different gender of distractors and targets than for items with same gender of the pictorial alternatives, indicating that the nouns were predicted by their gender category.

Within the experimental studies outlined above, the effects of gender markings provided in isolation (either carried by an article or an adjective) on eye-movements in noun-phrase-picture matching have been investigated. However, the listener is often provided with more than one predictively valuable piece of linguistic information within one phrase. In the German example given above (*ein^MASC or NEU^ netter^MASC +^^ANIMATE^ Hund^MASC + ANIMATE^* ‘a nice dog’), the lexical gender feature of the article indicates that the next upcoming noun should be from the masculine category. This prediction is subsequently confirmed by the gender feature carried by the following adjective. In addition, the adjective points to a noun from the semantic category ANIMATE. Since three distinguishable pieces of linguistic information may be used to predict the lexical features of an upcoming noun, the question arises whether and how they interact in online noun phrase decoding.

However, while many theoretical/conceptual and experimental studies have been published as to the interaction of linguistic information in the assignment of semantic/thematic roles to sentence constituents (e.g., Year, 2005; MacWhinney, 2008; Borovsky et al., 2013), up to now little is known about the interaction of linguistic information in the decoding of the noun phrase itself.

As far as we know, the three studies by Hopp (2013), Hopp and Lemmerth (2016), and Lemmerth and Hopp (2017) outlined within the introduction section provide the only evidence available until now for the predictive use of gender information in noun-phrase comprehension of German and only one of these studies directly addresses the population we are particularly interested in (monolingual German primary school children). Hence, the first aim of our study was to verify the predictive value of gender information as observed by Lemmerth and Hopp (2017) using a modified analytical approach. Instead of first looks at the target, we used binary coding whether the correct picture was fixated or not as a dependent variable and related this measure fixation probability to the time course of stimulus presentation. For example, we analyzed whether and when the probability of target fixations changed after presenting predictively usable bits of linguistic information. A comparable approach has been used for the investigation of semantic cue processing by Borovsky et al. (2013).

In addition, following the results reported by Hopp (2013), Hopp and Lemmerth (2016), and Lemmerth and Hopp (2017), we aimed to investigate the impact of gender agreement cues on noun phrase processing in more detail. Therefore, we adopted noun phrases as used by Lemmerth and Hopp (2017) in condition 2 (indefinite article + adjective + adjective + noun, (*e.g., ein kleiner gelber Eimer*) but introduced the following modifications:

(1) Instead of indefinite articles from the ambiguously gender marked masculine and neuter class (as used by Lemmerth and Hopp, 2017) the noun phrases we presented for picture matching were all selected from either the masculine or the feminine class (e.g., target *ein^MASC^ schön-er^MASC^ Löwe^MASC^* ‘a beautiful lion’; *eine^FEM^ gelb-e^FEM^ Katze*^FEM^ ‘a beautiful cat’). On account of this modification, both lexical precursors of the noun (article and adjective) provided predictively usable gender information, since in German the indefinite articles accompanying nouns from these classes are not homophonic. Thus, in contrast to previous studies (e.g., Hopp, 2013; Melançon and Shi, 2013; Hopp and Lemmerth, 2016; Lemmerth and Hopp, 2017) articles and adjectival suffixes provided converging information which could trigger a conjoint effect on noun prediction. For example, we believed that initial predictions based on the gender class of the article could lead to an increase of target fixation probability but remain below ceiling and the second gender cue from the adjectives could lead to a further increase of target fixation probability above the level already reached after processing the article.
(2) The noun phrases used by Hopp (2013), Hopp and Lemmerth (2016), and Lemmerth and Hopp (2017) only contained color adjectives (*gelb* ‘yellow’, *grün* ‘green’, *rot* ‘red’) or adjectives signifying size (*groß* ‘big’, *klein* ‘small’). With this restriction, the authors presumably aimed to avoid semantic relations between particular adjectives and nouns, which could have had an additional predictive effect interfering with the gender information, e.g., *ein gefährlicher Tiger* ‘a dangerous tiger’ presented with a distractor picture from a different gender category but depicting e.g., a mouse (*Maus*^FEM^). In this case, the semantic feature of the adjective could lead to predictive looks onto the target picture irrespective of the gender information provided by the adjective’s suffix. Alternatively, gender information provided by the suffix of the adjective could enhance the predictiveness of the gender information by providing converging semantic information from the adjective’s stem. This “cue coalition” of semantic and morpho-syntactic information might lead to a stronger influence of the gender cue in semantically related as opposed to unrelated adjective-noun combinations. Even the seemingly indifferent adjectives used by Hopp (2013) and Hopp and Lemmerth (2016) could have had this kind of impact on target fixation probabilities, e.g., the relation between *yellow* and *cheese* as in the example provided in Hopp (2013) might be considered closer than between *yellow* and the distractor pictures (e.g., *cupboard*, *helmet*). It has already been shown in visual-world studies that semantic information can influence fixation probability on target pictures and semantically related distractor pictures (e.g., Borovsky et al., 2013).

In order to explore this issue, we presented adjectives from a range of 12 and aimed to control for a semantic relation between the adjectives and the succeeding nouns. For that purpose, in 13 of the 30 trials in each condition (informative and uninformative) the semantic features of the adjectives pointed probabilistically to one of the two pictures (e.g., the adjective *kleiner* [small] in a trial depicting *Tiger* ‘tiger’ and *Vogel* ‘bird’). For these semantically related adjective-noun combinations, the gender cue provided by the gender suffix of the adjectives converged with the semantic information represented by the adjectival stem because both cues pointed to the same target picture. Introducing this additional distinction between semantically related and unrelated adjective-noun combinations we aimed to explore, whether the gender cue caused additional anticipatory effects in those cases in which the target was probabilistically predictable on semantic grounds.

If the experimental paradigm as outlined in detail below could be shown to reflect relevant aspects of online gender processing in monolingual German primary school children, it could be taken to explore gender-processing in other German-speaking populations in follow-up research. For example, for school age children with Specific Language Impairment (SLI) gender processing has been identified as a potential area of vulnerability (Clahsen, 1991; Anderson and Souto, 2005; Orgassa and Werman, 2008; Varlokosta and Nerantzini, 2013). As a result, it can be expected that at least some of these children show different eye-movement patterns compared to typically developing peers. Yet, not much is known about online gender processing in auditory comprehension of German children with and without SLI.

## Materials and Methods

### Participants

A total of 32 monolingual German speaking children participated in the study (13 females, 19 males, mean age = 9;1 [years;months], range = 8;2 to 9;8, 3rd or 4th grade). Even though gender processing might already be observable in younger children acquiring German, the participants were selected from this age range because of two reasons: Firstly we aimed to compare our results with those reported by Lemmerth and Hopp (2017), who investigated gender processing in monolingual German children in this age category. Secondly, in a follow-up research study we intend to use the experimental paradigm tested here in order to explore gender processing in 3rd and 4th grade children with *developmental language impairment* and compare the performance of this population with the performance of a aged-matched control group.

The children received an amount of 10 € for participation. Informed parental consent was obtained for all participants. Likewise, the children were informed about the general objectives and contents of the study and agreed to participate. The study was approved by the Ethical Committee of the University Hospital in Aachen.

According to short interviews with the parents, none of the children had a history of neurological, attentional or developmental disorders and all showed normal or corrected to normal vision and hearing. Mean non-verbal IQ as measured by the Colored Progressive Matrices (CPM, Bulheller and Häcker, 2002) was 110 ± 14. Parents were asked to complete the Child Behavior Checklist (CBCL/6-18R; Döpfner et al., 2014) indicating that none of the children exhibited behavioral difficulties (mean t-score: 49.9 ± 7.5).

To rule out developmental language disorders, we administered the Test of Reception of Grammar (TROG-2, Bishop, 2003; German Version: Fox, 2016), assessing auditory comprehension for syntactic structures using sentence picture matching tasks. All children performed at an age-appropriate level in the mean t-score: 52.4 ± 8.0. In addition, reading disorders were excluded by means of the *Ein Leseverständnistest für Erst- bis Sechstklässler* (ELFE 1-6; Lenhard and Schneider, 2006) wherein all children performed at an age appropriate level as well (mean *t*-score: 58.9 ± 8.3). These individually administered tests took 30–40 min.

### Picture Selection

**FIGURE 1:**
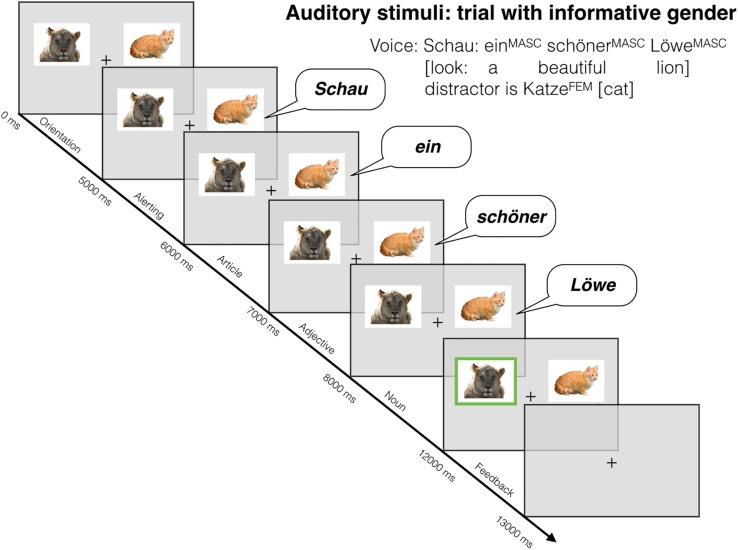
Example of an informative trial.

Colored photographs were selected from an open source web library^1^, depicting prototypical objects or animals which could be named using a monomorphemic German noun (e.g., *Teller* ‘plate’, *Tiger* ‘tiger’, *Hase* ‘hare’, cf. Figure 1). The nouns were controlled for frequency^2^. We chose the category lemma to create a lexicon and selected the corpus MannMIn, which gives information on a word’s frequency among a million words for spoken and written language. The value < 10 was used to classify words with low frequency, words with a value of 10–100 were assigned to the category medium frequency and words with a value > 100 were regarded as high frequency words. It was assumed that German 3rd and 4th grade children were able to name the nouns. Prior to the eye-tracking experiments, it was assessed whether the participants did in fact use the intended nouns in a naming task containing the photos. In the rare cases of an incorrect or semantically related response (e.g., *Kaninchen* ‘rabbit’ instead of *Hase* ‘hare’), the target noun was named orally by the examiner.

### Language Stimuli

The language stimuli were noun phrases consisting of an indefinite article, an inflected adjective and a monomorphemic, one- or two-syllable noun (e.g., *ein-e klein-e Tasse* ‘a small cup’). They were presented auditorily using speaker systems.

As outlined above, in German noun phrases the gender features of all inflected words are controlled by the noun. By implication, the gender feature of a noun can often be predicted by the gender category of a preceding article and/or adjective. For example, the inflectional features of the article *ein-e* and the adjective *klein-e* indicate that the gender category of the following noun must be feminine (e.g., *ein-e klein-e*…*Tasse*^FEM^ ‘a small cup,’ *Gabel*^FEM^ ‘fork’…) and not masculine (e.g., *Teller*^MASC^ ‘plate,’ *Löffel*^MASC^ ‘spoon’). In contrast, the inflectional forms of the same article and adjective lexemes within a noun phrase indicating masculine would have to be *ein klein-er (e.g., ein kleiner Teller* ‘a small plate’). Thus, consideration of gender agreement rules allow to restrict the search space for a noun following the sequences *ein klein-er* (noun)^MASC^ and *ein-e klein-e* (noun^FEM^), because only nouns from one gender category have to be compared with the phonological and pictorial input.

Within the noun phrase picture matching tasks used in our experiment, this structural property of the German language allowed to distinguish informative and uninformative trials as introduced by Johnson (2005) for Dutch (cf. Figure 1, for an example of an informative trial). The complete item sample comprised 30 informative and uninformative article-adjective-noun sequences each (60 trials in total). Trials were presented in different, randomized successions to each of the participants in order to avoid sequencing effects for particular items.

#### Selection of the Nouns

To generate the 60 noun phrases, 30 different depictable monomorphemic nouns were used, 13 from the feminine and 17 from the masculine gender. Nouns from the neuter category were not included, because in German the indefinite articles are homophonic for the neuter and the masculine gender class (cf. Table 1). The uneven distribution of masculine and feminine nouns can be explained as follows: An originally larger item set consisted of 68 items, 34 each from the informative and the uninformative condition. Herein, 17 of the nouns in both conditions were selected either from the masculine or from the feminine category. As outlined above each item consisted of two pictures and a noun phrase corresponding with one of the pictures. In order to explore whether the two pictures attracted visual attention to different degrees irrespective of the language stimulus (which could add unwanted noise to the eye-movement pattern observed during the noun-phrase picture matching task), we analyzed eye movements onto the two pictures during the first 5000 ms after stimulus onset. During this period the participants had time to look at the pictures without listening to a verbal stimulus. An item analysis revealed that some of the pictures attracted significantly more overall fixation time than their pictoral neighbors, presumably due to their particular visual or thematic attractiveness (e.g., *Koffer* ‘suitcase’ paired with Teller ‘plate’; *p*-value < 0.05 with binomial test). These items remained unconsidered for further data analysis. This revision of our item set resulted in the unequal distribution of nouns from the masculine and feminine gender class (see Supplementary Tables S1, S2, providing detailed information on the language stimuli that were used).

**TABLE 1:** Gender agreement in German noun phrases containing a definite or an indefinite article.

	**Article**	**Adjective**	**Noun**
**Noun phrases^NOMINATIVE_SINGULAR^ containing definite articles**			
Masculine	der [the]	nett-e [nice]	Igel [hedgehog]
Feminine	die [the]	nett-e [nice]	Biene [bee]
Neuter	das [the]	nett-e [nice]	Huhn [chicken]
**Noun phrases^NOMINATIVE_SINGULAR^ containing indefinite articles**			
Masculine	ein [a]	nett-er [nice]	Igel [hedgehog]
Feminine	ein-e [a]	nett-e [nice]	Biene [bee]
Neuter	ein [a]	nett-es [nice]	Huhn [chicken]

Length (one or two syllables) and frequency (see footnote 2) of the nouns were balanced across informative and uninformative trials. Furthermore, nouns with phonological endings pointing to a specific gender category were balanced across informative and uninformative trials as well. Each noun was used once or twice as a target within the 60 trials.

#### Selection of the Adjectives

One- or two-syllable inflected adjectives from a set of 12 preceded the 30 nouns within the noun phrases. Each adjective appeared equally often within the informative and uninformative trials. The distractor pictures were not selected in order to contrast with the semantic content of the adjectives. For example, the noun phrase *a small cup* was not accompanied by a distractor photo depicting a *large* cup but by a distractor depicting a small *plate*.

In order to control for semantic relations between an adjective and the two depicted objects presented within a trial, we conducted a pilot study with 23 students. We presented each adjective together with the two pictures as they were assembled within the 60 trials of the eye-tracking experiment and asked the participants to judge whether one of the two pictures “fitted” better with the adjective than the other picture. For example, we expected the participants to judge *wild* ‘wild’ being more closely related to *Löwe* ‘lion’ than to *Käfer* ‘bug’ but *Robbe* ‘seal’ and *Affe* ‘monkey’ being indifferent with respect to the semantic relations to the adjective *groß* ‘big’. For 23 of the 60 adjective-picture assemblies, more than 80% of the students judged one picture to be more closely related to the adjective than the other and we assigned these adjective-picture (accordingly adjective-noun) pairings to be semantically related. Precisely, 11 semantic cues were included in the informative gender condition and 12 semantic cues were included in the uninformative gender condition.

Within the interactive review process one reviewer scrutinized the plausibility of the semantic relatedness judgments observed in our pilot study. For example, according to the ratings of the participants, a lion is more likely to be old than a hare (Supplementary Table S1, Item 12), a hare is more likely to be nice than a lion (Item 9) but a boy is not more likely to be nice than a lion (Item 15). We admit that our own semantic intuitions were not always congruent with each other and with those of the student participants. Obviously, the semantic intuitions underlying the ratings for some of the items were quite subtle, associative and they cannot easily be operationalized in terms of semantic features. In addition, the judgments required quite complex semantic decisions, since always two nouns had to be related to one adjective and the adjective itself might be interpreted by adding varying semantic connotations on the compared noun. Thus, participants might have spontaneously and intuitively assumed, that lions have a much longer expectancy of life than hares and are thus more plausibly assumed to be old than hares or that boys and lions are equally often not nice as expressed within the frequent collocation “bad boy,” for example. Of course, these *ad hoc* explanations are speculative since we did not ask the participants to substantiate their judgments.

Within the trials with uninformative gender markings, the semantic information provided by the adjectives could trigger predictive looks onto to the target picture even though no gender information was provided. In contrast, within the trials with informative gender markings, the semantic information provided by the adjective converged with the gender information represented by the suffix of the adjective, because information from both sources pointed to the same target picture. Introducing this additional distinction between semantically related and unrelated adjective-noun combinations, we aimed to explore whether the gender cue caused additional anticipatory effects even though the target was probabilistically predictable on semantic grounds only.

#### Selection of the Articles

The indefinite article in each noun phrase was either the two-syllable word *ein-e^FEM^* or the one-syllable word *ein^MASC/NEU^* ‘a’. Indefinite articles were used instead of definite ones (e.g., *der, die, das* ‘the’), because in German adjectives succeeding definite articles do not change their form with respect to the gender category of the noun (cf. Table 1). Thus, using definite articles would have made it impossible to provide distinctive gender information via the inflectional form of the adjectives. In addition, the paradigm of the nominative definite articles as provided for masculine and feminine gender is characterized by morpho-phonological ambiguity with respect to number, since *die* can either indicate *feminine singular* (e.g., *die^FEM/SING^ Katze^FEM/SING^*) ‘the cat’ but also, for example, *masculine plural* (e.g., *die^MASC/PLUR^ Hasen^MASC/PLUR^*) ‘the rabbit’. This ambiguity does not exist for indefinite articles which cannot take plural forms in German.

### Procedure

#### Picture Presentation and Alerting

At the beginning of each trial, two photographs were presented. In order to allow the children to become familiar with the pictorial information, the photos were presented 5000 ms prior to the onset of the language stimulus. In order to exclude side effects, target pictures were shown equally often on the left and on the right side of the screen. At the end of this phase, an auditory alert followed: *Schau* ‘look’ to attract the child’s attention to the upcoming language stimulus. No items needed to be dropped due to problems in attention or orientation.

#### Stimulus Presentation

The language stimuli were provided by speaker systems. The participants could choose the loudness of the stimulus presentation according to their preference. The noun phrases were recorded as a whole from a native, dialect-neutral female speaker, articulating distinctly with a natural speech rate of 3 syllables per second. Afterward, the noun phrase records were split up into single word records. These were successively played word by word in fixed intervals of 1000 ms each. Since the presentation of the single words took less than 1000 ms, short pauses appeared between the words within a noun phrase.

Thus, in each trial the participants had 2000 ms of time to process the article and the adjective before the first phoneme of the noun was presented, which is about 300 ms longer than in the study of Hopp (2013). The language stimuli resulting from this mode of presentation sounded similar to the output voices provided by route navigation systems. We considered this deceleration of input rate appropriate for primary school children who might need more time to process gender information due to less proficient processing routines.

In addition, as outlined in the introduction, an aim of the present study was to collect baseline data with typically developed children according to which children with SLI/Developmental Language Disorder (DLD)^3^ can be compared in a follow-up research project. As shown by Montgomery (2005), sentence comprehension of school age children with SLI can improve, if the stimuli are presented at an input rate slowed down for about 25% in comparison to a normal rate. If indicators of gender agreement processing would be observable under decelerated input conditions, we could proceed to successively increase input rate in order to explore the effects of these alterations onto the eye-movement patterns observable in typically developed children and children with SLI/DLD.

#### Consolidation and End of Trial

To indicate the end of the response/consolidation phase, the target picture was highlighted by a green frame and the children were instructed not to look away from the target picture, before the green frame was presented. In addition, the green frame was provided to support the children maintaining alertness and compliance during the whole experiment. The following trial was started manually as soon as the child focused on the fixation cross in the middle of the screen. Including repeated calibration, instruction and the 60 trials, the duration of the experiment did not exceed 30 min. A typical session took approximately 25 min.

#### Instruction

Each child was instructed using a video in which the course of the experiment was demonstrated verbally and visually and in which examples were given. Detailed instruction was provided particularly on how to perform the eye-tracking task (e.g., avoidance of body movements as well as eye movements not directed to the screen). Children were explicitly invited to look at the picture they assumed to be the target as soon as possible and even if the presentation of the language stimulus was not finished yet. Apart from the standardized instruction, the children were allowed to ask questions at any time. No instruction referring to grammatical gender was given.

### Technical Details

Auditory stimuli were displayed in CD quality. Picture size was 300 × 400 pixels, presented in front of a gray screen (RGB 180, 180, 180). Right-eye movement measures were recorded using the EyeLink 1000 desktop-mount video-based system (SR Research), which includes a camera with high temporal and spatial resolution. The system operates contact free. The visual stimuli were presented on a 22′′ TFT screen with a resolution of 1680 by 1050 pixels and a refresh rate of 120. Children’s correct head position was ensured by a chinrest. The participants did not have to push any buttons. Accuracy of eye movement recording was established with a 9-point calibration at the beginning and four times during the experiment. The targets for calibration (small circular targets) were shown in a validation procedure again for double-check. A fixation cross was used to ensure the children’s proper and constant sitting position during and between the trials.

### Approach to Data Analysis

The data we collected consisted of records of the participants’ gaze on one of the two pictures while they were listening to the noun phrases. Raw fixation data were converted to fixation-based matrices (edf-files) using the software DataViewer (SR Research). Fixation data of all participants (*n* = 32) were assembled. Only fixations on the two pictures were included in the analyses.

As dependent variable, we used binary coding whether the correct picture was fixated (1) or not (0). Generalized linear mixed effect models (GLMM) with a logit link and a binomial distribution of residuals with subject and item-number as random factors were computed in R (R Core Team, 2016) with lme4 (Bates et al., 2019). In the analyses, the random factor “item-number” was nested in the random factor “subject.” Possible predictor variables for the fixation probability were the continuous variable TIME (change in time in steps of 50 ms), the factor GENDER (informative/uninformative) and the factors SEMANTIC CUE (semantic relation between adjective and noun YES or NO).

The predictor variable TIME was centered around the mean for each specifically analyzed time component of the experiment (alerting, article, adjective and noun^first^ ^1000^ ms, see below). For each of these time components, we conducted a separate model and removed non-significant predictor variables (only GENDER and/or SEMANTIC CUE) from GLMMs unless they were constituent parts of significant interaction terms. Different models were compared with likelihood ratio (LR) tests to find the best model in terms of fit and sparseness for each phase. The summary of the best models for alerting, article, adjective and noun is prepared with the R-package sjPlot (Lüdecke and Schwemmer, 2018). *P*-values for the predictors and the number of fixations are included in the model (observations).

Graphs were created with ggplot2 (Wickham, 2009). Smoothed means are presented in the plots using a natural spline function with a moderate degree of smoothing. Fixation probability is calculated on the time line in steps of 50 ms.

## Results

**FIGURE 2:**
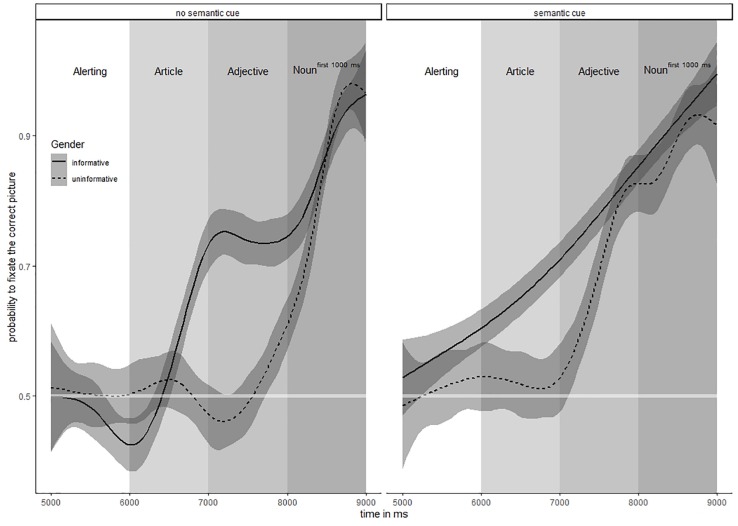
Time course of target fixations – the lines represent the relation between the number of fixations on the correct picture and the total number of fixations (fixation probability correct picture). A fixation probability would be 1 if all fixations were on the target. A comparable number of fixations on correct and incorrect pictures (fixation probability 0.5) is expected in the first 6000 ms, wherein no information is given and the children were free to look around.

Results of the analysis are depicted graphically in Figure 2. In the figure, the probability of fixations onto the target is plotted for uninformative trials (dotted line) and informative trials (solid line). Trials with semantically unrelated adjective-noun combinations are represented on the left side of the graph and semantically related combinations on the right.

In order to illustrate the time course of target fixations, four different time components are distinguished, namely alerting phase (white column), article presentation phase (bright gray column), adjective presentation phase (medium gray) and noun^first^ ^1000 ms^ presentation phase (dark gray).

To confirm the effects suggested by the visual inspection of the time course plots, statistical information is given in Table 2. Within the table, the models with the best fit are presented separately for the four time components.

**TABLE 2:** Results of the GLMM analysis of fixation probability to the correct picture for the time components alerting, article, adjective and noun^first 1000 ms^.

	**Alerting**		**Article**		**Adjective**		**Noun**	
***Predictors***	***Odds ratios***	***CI***	***Odds ratios***	***CI***	***Odds ratios***	***CI***	***Odds ratios***	***CI***
(Intercept)	1.016	0.812 – 1.270	1.708^∗∗∗^	1.447 – 2.016	4.497^∗∗∗^	3.424 – 5.908	16967.385^∗∗∗^	4968– 57943
Time	1.000	1.000 – 1.000	1.002 ^∗∗∗^	1.001 – 1.002	1.000	1.000 – 1.001	1.009^∗∗∗^	1.007 – 1.010
Gender			0.645^∗∗∗^	0.513 – 0.810	0.253^∗∗∗^	0.187 – 0.340	1.418	0.455 – 4.424
Time × Gender			0.998^∗∗∗^	0.998 – 0.999	1.001	1.000 – 1.001	1.004^∗∗∗^	1.002 – 1.006
Semantic cue					1.420	0.998 – 2.021	0.528	0.136 – 2.049
Semantic cue × Gender					2.337^∗∗∗^	1.452 – 3.761	0.964	0.183 – 5.072
Semantic cue × Time					1.002^∗∗∗^	1.001 – 1.002	0.995^∗∗∗^	0.993 – 0.997
**Random effects**								
σ^2^	3.29		3.29		3.29		3.29	
τ_00 ITEM–NUMBER:SUBJECT_	2.69		1.41		1.65		85.88	
τ_00 SUBJECT_	0.19		0.00		0.16		0.00	
ICC _ITEM–NUMBER:SUBJECT_	0.44		0.30		0.32		0.96	
_SUBJECT_	0.03		0.00		0.03		0.00	
Observations	2204		2703		3392		3672	
Marginal *R*^2^/Conditional *R*^2^	0.000/0.467		0.031/0.322		0.107/0.424		0.073/0.966	

*Subjects and item-numbers were included in the model as random effects, wherein the variable item-number was nested in the SUBJECT variable. The formulas of the models were as follows: Alerting: Target fixation probability ∼ Time + (1|subject/item-number); Article: Target fixation probability ∼ Time ^∗^ Gender + (1|subject/item-number); Adjective: target fixation probability ∼ Time ^∗^ Gender + Semantic cue ^∗^ Gender + Semantic cue ^∗^ Time + (1|subject/item-number); Noun^*first*^^1000*ms*^: Target fixation probability ∼ Time ^∗^ Gender + Semantic cue ^∗^ Gender + Semantic cue ^∗^ Time + (1|subject/item-number). *T*_00_ = between individual variance; CI = Confidence Interval; ICC = Interclass Correlation Coefficient; Marginal and conditional *R*^2^ are the coefficients of discrimination. The intercept refers to the mean of the first 50ms within the modeled time frame; ^∗^*p* < 0.05; ^∗∗^*p* < 0.01; ^∗∗∗^*p* < 0.001.*

### Alerting Component

During the alerting component, no language stimulus and thus no relevant linguistic information was provided. Consequently, probability of target fixations falling on each of the two pictures was not assumed to differ significantly from chance level (0.5 in a two-picture choice) in all experimental conditions and during the whole period (1000 ms). Neither TIME nor the linguistic factors GENDER or SEMANTIC CUE or interactions of these factors could explain the variance of target fixations. These observations confirmed that the children did not prefer the target in any of the experimental conditions or any subperiod of this time component. The best model only included the non-significant predictor TIME (conditional *R*^2^ = 0.467), pointing to the fact that a large amount of the eye movement behavior was reducible to random individual differences or differences in items per subject (see τ_00_ in Table 2). Hence, we were able to analyze the further time course of target fixation probabilities from a baseline which was homogenous and unbiased in all experimental conditions.

### Article Component

During the article component, the gender agreement information was provided for the first time – but within the informative trials only. Thus, the probability of target fixations was expected to rise significantly above 0.5 in this experimental condition, while it should remain within the confidence limits of 0.5 in the uninformative trials. No effect of SEMANTIC CUE was assumed to appear, since the adjective providing information on semantic relatedness with the noun was not presented yet. Furthermore, as the relevant morpho-syntactic information was imparted by the presence or absence of the second morpheme (*ein* versus *ein-e)*, it was not provided from the very beginning of this component. As a consequence, we expected the probability of target fixations to increase within the 1000 ms period representing this component.

These expectations were confirmed: As indicated by the time course plots in Figure 2, the probability of target fixations for the uninformative trials (dotted line) remained at a chance level (probability 0,5) and increased markedly above 0.5 for the informative trials (solid line). The effects suggested by visual inspection were confirmed by the statistical analysis (best model conditional *R*^2^ = 0.322; Table 2), wherein eye movement behavior was especially explainable by the added predictor variable GENDER (cf. Table 2: reduced τ_00_ and ICC of the article-model compared to the alerting-model). The intercepts indicate a significant difference from 0.5 probability which can be traced back to an increase of fixations on the target pictures. The effect for TIME indicates that the fixation probability in favor of the correct picture increased over time (main effect time). But only for the informative trials we found an increasing amount of target fixations (interaction TIME X GENDER).

In summary, these observations indicate an increase of target fixation probability for the informative items during the article component. Hence, as soon as the article was presented, participants favored the target picture according to linguistic predictions based on gender processing.

### Adjective Component

Within the adjective component, two additional predictively usable pieces of information were provided either in isolation or in convergence. We expected target fixation probability to increase during the adjective component due to significant effects of the factors GENDER, SEMANTIC CUE and TIME, which was confirmed by statistical analysis. As expected, the best model for the adjective component included the predictors TIME, GENDER and SEMANTIC CUE (conditional *R*^2^ = 0.424).

Our assumptions as to the effects observable during this time component were intricate (at least threefold). First, for all informative trials a second gender agreement information was presented by the second morpheme of the adjectives (e.g., *ein*^MAS^ *klein-er^MAS^* ‘a small’ …versus *ein-e^FEM^ klein-e^FEM^*…). Participants‘ initial predictions concerning a particular gender category of the following noun could now be supported with this converging morpho-syntactic evidence and we assumed that this might lead to a further increase of target fixation probability. Since gender information was not provided until the suffix could be processed, we expected an interaction of TIME X GENDER to appear in this case. If our hypothesis was true, the isolated effect of this additional gender cue could specifically be observed considering the informative trials with no semantic relation of adjective and noun (solid line in the left part of Figure 2).

**FIGURE 3:**
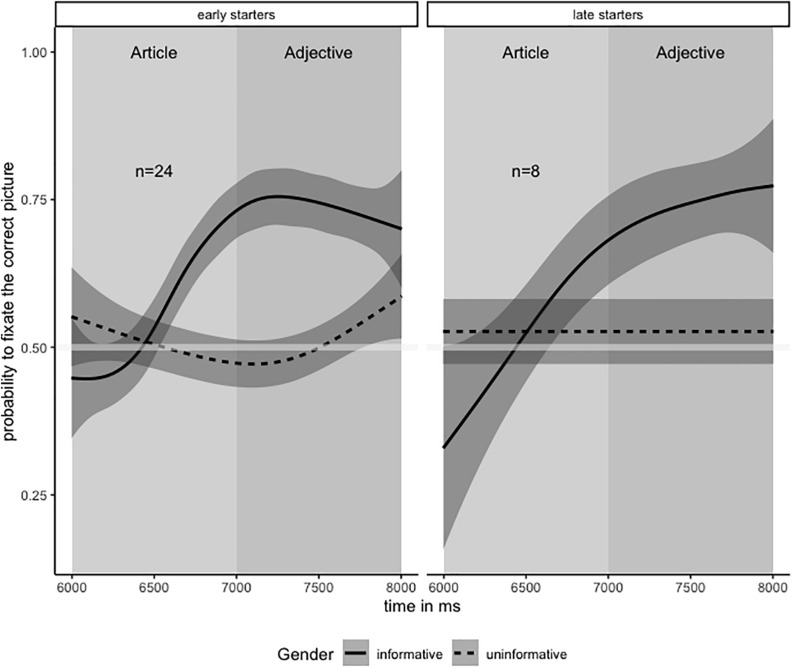
“Late” and “early starters.”

This specific assumption could not be confirmed. Contrary to our expectations, the additional gender cue did not cause any further increase of fixation probability on the target picture, which can be observed considering the solid flat line in the left part of Figure 2. In other words, the additional gender agreement information did not further help the participants to find the right picture. Accordingly, the *p*-value for the interaction TIME X GENDER did not reach significance. Since the missing effect of the adjectival gender information conflicted with our theoretical expectations, we wondered whether it was observable for all of the 32 participants. Therefore, we conducted a *post hoc* analysis revealing the individual slopes of fixation probabilities during the adjective component. Within the adjectives, we compared the number of correct fixations for each participant in the first 500 ms with the last 500 ms using an exact binomial test in the software package R. Depending on the result (significant difference or not), the children were divided into two groups. The results are depicted in Figure 3. Unsurprisingly, most of the children (*n* = 24/32) showed the flat slope line of target fixations as outlined above for the complete sample. These children gained a target fixation probability of 0.75 when provided with the gender information of the article and were not reassured additionally by the converging adjective gender agreement information (right part of Figure 3). However, a minority of the children (*n* = 8/32) showed eye movement patterns differing from the majority. For these children, target fixation probabilities within the informative trials only reached levels below average during the article component but further increased during the adjective component (left part of Figure 3). At the end of the adjective component, these “late starters” reached the same above chance level of target fixation probability (approximately 0.75) as it was observed for their faster responding peers. The late starting pattern could either be traced back to an impact of the gender information provided by the adjectives or to a retarded response to the gender information provided by the articles.

Anyway, the eye-movement patterns observable during the article and the adjective component appeared to differ individually. Eventually, the late-starting pattern reflects a less proficient use of gender agreement information relying either on more time for psycholinguistic processing or on more converging linguistic information pointing to the same structural interpretation. Admittedly, our observations should be interpreted with caution since the comparison of samples with considerably differing sizes (8–24) could be misleading. Further research would be needed to clarify these unexpected preliminary findings.

Secondly, for all trials with a semantic relation between adjective and noun (irrespective of the gender cue being informative or not), an additional non-grammatical predictor was introduced, represented by the semantic features of the adjective stem. The isolated effects of this semantic information could specifically be observed in trials with uninformative gender agreement information (dotted line in the right part of Figure 2). The semantic information provided in isolation clearly had an influence onto target fixation probabilities (dotted line of the right part of Figure 2). The statistical analysis revealed a significant interaction of TIME X SEMANTIC CUE (Table 2).

Thirdly, as to the informative trials (solid line in the right part of Figure 2), the semantic information converged with the first and the second gender agreement information as it pointed to the same picture. Our question here was, whether this convergence of linguistic information would have an influence on the predictive strength of the gender agreement cue provided by the adjectives. Here, a significant interaction of GENDER X SEMANTIC CUE was detected. This interaction is the result of different starting points for the informative and uninformative trials (see right part of Figure 2; dotted line 0.5 and solid line 0.7). But both lines end up at same fixation probability (0.8), meaning that trials providing semantic information only compared with trials providing semantic and gender information reach the same fixation probability for the target picture.

### Noun^first^^1000*ms*^ Component

During this final time component, the phonological form of the noun was presented. Consequently, the participants did not need to predict the target picture any more on morpho-syntactic and/or semantic grounds but could select it unambiguously from lexical access to the target lexemes of the nouns. As expected, the probability of target fixation approximated the 1.0 level for all experimental conditions during this time component (cf. Figure 2). The strong effect of the intercept indicates that the task was solved unambiguously during this component (fixation probability > 0.95 at the end). The best model explaining variance during the noun^first^ ^1000 ms^ component included significant effects for predictors TIME and interaction of TIME X GENDER and TIME X SEMANTIC CUE (conditional *R*^2^ = 0.966). These effects could be traced to the fact that in trials with informative gender (as well as in trials with a semantic relation between adjective and noun) the final increase of target fixations induced by the nouns’ phonemes started from a much higher base level (roughly 0.80 in informative trials). Consequently, for morpho-syntactically and semantically uninformative trials the slope had to increase steeper in order to reach the final probability level (0.95) for target fixations. However, with regard to the eye movement pattern observed during the noun^first^ ^1000 ms^ component it is of course not excludable, that they not only reflect processing of the noun itself but spillover effects from the previous word (i.e., the adjective).

## Discussion

The results of the present study confirm findings observed for children from other gender-marking languages (Lew-Williams and Fernald, 2007; Melançon and Shi, 2013; Brouwer et al., 2017), for German speaking adults (Hopp, 2013; Hopp and Lemmerth, 2016) as well as for monolingual German speaking primary school children (Lemmerth and Hopp, 2017). It could be shown that German 3rd and 4th graders use gender information in order to predict the noun according to its agreement with preceding gender marked words.

For the majority of the children the predictive effect of gender agreement was observable immediately after the article was presented, as was the case for the adult participants in the studies of Hopp (2013) and the primary school children in the study of Lemmerth and Hopp (2017). However, predictions based on the first gender cue were not enhanced by converging gender information provided by the adjectives. Accordingly, these children did not update the certainty of their expectations due to the second gender cue, even though the probability of target fixations was far below 100% at this point of stimulus presentation. Maybe, the second gender cue remained more or less unconsidered, because it was from the same linguistic source as the first cue (i.e., gender agreement) and thus was not taken to add a new bit of predictively relevant information.

However, as revealed by *post hoc* analysis for a minority of the children, the probability of target fixations in the case of the informative trials markedly increased during the adjective component. We propose to call these children “late-starters” because we assume that they probably needed a few hundred milliseconds more time to process gender information provided by the article. Hence, even though the influence of gender information was not obvious before the adjective component was presented for this subgroup, we assumed this effect to originate from the article component. Alternatively, the processing routines of these children might have depended on converging morpho-syntactic evidence from the same source (gender) and could not be triggered by an isolated, though valid gender cue. These alternative assumptions need to be clarified by further research.

In addition, not only gender information but also semantic information as provided by the stems of the adjectives influenced the probability of fixations onto the target pictures. This assumption can most distinctly be derived from the eye movement patterns in trials which did not provide predictively relevant gender information but allowed to predict the target picture from the semantic features of the adjective. Within these trials, target fixations remained at chance level during the article component but significantly increased during the adjective component. This effect parallels the observations reported by Borovsky et al. (2013) for sentences and confirms that semantic relations of succeeding words can be used to predict the continuation of a sentence. Consequently, semantic relatedness between adjective and noun must be considered a potentially relevant factor influencing eye movements during noun phrase picture matching.

Another interesting finding in the adjective component is that semantic information in combination with gender information did not result in a higher prediction level compared to stimuli with semantic information only. In addition, semantic cues resulted in higher levels of fixation probability (0.8) than gender cues (0.7).

Finally, we focused on the noun^first^ ^1000 ms^ component, during which the phonological form of the target noun itself was introduced. Since target probabilities significantly increased and finally reached ceiling level (1.0) only during this time component, the children obviously used this phonological information in order to confirm their predictions and to finally select the target picture undoubtedly. Interestingly, this was true even for the informative trials, in which the target could be predicted unambiguously from gender agreement alone. Despite the high probability of image A, the possibility is kept open that image B could also be named. This observation is in line with the results reported by Hopp (2013) and by Hopp and Lemmerth (2016) for monolingual German-speaking adults.

## General Discussion

In summary, the experimental design and psycholinguistic assumptions we adopted to explore gender processing in online auditory comprehension of primary school children turned out to be generally conclusive and promising to justify further research on that matter.

To clarify our results in follow up studies, psycholinguistic processing models should be adopted that account for dynamically varying influences of gender agreement cues provided either in isolation or in combination with other bits of linguistic information from different sources (grammatical, semantic, phonological) during well defined time course components. For example, it would be interesting to explore target fixation probabilities for trials in which the gender cue points to a different interpretation than the semantic cue (e.g., *ein-e^FEM^ brav-e^FEM^ Spinne^FEM^* ‘a good spider’, distractor: *Hund* ‘dog’^MASC^). Evidence for an interaction between semantic and gender information is provided by several ERP reading studies, demonstrating that gender information has a stronger effect on event-related potentials when the noun is semantically unexpected (e.g., Wicha et al., 2004, for Spanish and Hagoort, 2003, for Dutch).

Evidence for an interaction between phonological and gender information is provided, for example, by the study of Dahan et al., 2000, cited in the introduction, demonstrating that phonologically based processing routines (i.e., cohort competition) can be strongly influenced by morpho-syntactic information (i.e., gender agreement).

In addition, the impact of varying rates and modes of stimulus presentation on gender processing need to be clarified. In the present study we presented the language stimuli rather slowly and with pauses after each word, which might have triggered the predictive use of gender information. As outlined in the introduction, we chose this slow mode of presentation with prospect to an assessment of children with SLI/DLD in a follow-up study, which we are preparing currently. It is widely held in speech language therapy and special education that children with developmental language disorders might take advantage of decelerated and prosodically over-structured input (Berg, 2006; cf. Cunningham, 2011). However, in order to play a role in normal communication, gender cues need to be usable even if the language stimuli would be presented with an average speed of presentation and without pauses between succeeding words.

Furthermore, the influence of gender agreement on other aspects of receptive grammatical processing, e.g., decoding of agent-object relations in topicalized sentences and relative clauses, could be explored using a similar eye-tracking design as we used in our experiments (cf. Adani et al., 2010; Belletti et al., 2012; Biran and Ruigendijk, 2015). For example, if the German sentence *Den^MASC–ACCUSATIVE^ Jungen^MASC–ACCUSATIVE^ küsst das^NEUT–NOMINATIVE^ Mädchen^NEUT–NOMINATIVE^* ‘as for the boy, the girl kisses him’ is presented together with the target and distractor picture showing a girl who kisses a boy, the rejection of the distractor could be influenced by the interaction of gender and case information provided by the definite article *den^MASC/ACCUSATIVE^*. Proficient listeners could use this morpho-syntactic cue to make early predictions about the unusual but grammatically well-formed succession of the thematic roles in German (i.e., object → action → agent). This mode of predictive top–down processing could be an important advantage in order to comprehend efficiently and effortlessly.

## Data Availability Statement

The datasets generated for this study are available on request to the corresponding author.

## Ethics Statement

The studies involving human participants were reviewed and approved by the Ethical Committee of the University Hospital in Aachen. Written informed consent to participate in this study was provided by the participants’ legal guardian/next of kin.

## Author Contributions

JC and TG conceived the idea behind the study and developed the theory. TG performed the analyses. IN carried out the experiments and collected the data. JC, TG, IN, and AB discussed the results and contributed to the final manuscript.

## Conflict of Interest

The authors declare that the research was conducted in the absence of any commercial or financial relationships that could be construed as a potential conflict of interest.

## Footnotes

1 www.pixabay.com

2 celex.mpi.nl

3 Bishop et al. (2017) proposed to use DLD as the preferred term for language problems and, in this way, distanced themselves from the exclusionary criteria in the definition of language disorder.
