# Supplementary material for: Online-Processing of Grammatical Gender in Noun-Phrase Decoding: An Eye-Tracking Study With Monolingual German 3rd and 4th Graders
Source: Front Psychol. 2019 Nov 15;10:2586. doi: 10.3389/fpsyg.2019.02586 (PMC6873886; doi:10.3389/fpsyg.2019.02586)
Supplement: Supplementary file 1 [file Table_1.docx]

Table S1

*An overview of the items used in the study.*

| **Item** | **Vocally presented phrase** | **Distractor noun** | **Category** |
| --- | --- | --- | --- |
| 1 | Eine große Ampel [a big set of traffic lights] | Muschel [shell] | no semantic cue, no gender cue |
| 2 | Eine zarte Nadel [a filigree needle] | Ampel [set of traffic lights] | no semantic cue, no gender cue |
| **3** | Ein bunter Apfel [a colourful apple] | Esel [donkey] | semantic cue, no gender cue |
| **4** | Eine kleine Muschel [a small shell] | Ampel [set of traffic lights] | semantic cue, no gender cue |
| **5** | Eine schöne Ampel [a beautiful set of traffic lights] | Nadel [needle] | no semantic cue, no gender cue |
| **6** | Ein braver Esel [a well-behaved donkey] | Apfel [apple] | semantic cue, no gender cue |
| **7** | Eine feine Butter [a delicate piece of butter] | Feder [feather] | no semantic cue, no gender cue |
| **8** | Eine gute Feder [a good feather] | Butter [butter] | no semantic cue, no gender cue |
| **9** | Ein lieber Hase [a nice hare] | Löwe [lion] | semantic cue, no gender cue |
| **10** | Ein neuer Junge [a new boy] | Hase [hare] | no semantic cue, no gender cue |
| **11** | Ein wilder Hase [a wild hare] | Junge [boy] | no semantic cue, no gender cue |
| **12** | Ein alter Löwe [an old lion] | Hase [hare] | semantic cue, no gender cue |
| **13** | Ein braver Hund [a well-behaved dog] | Mund [mouth] | semantic cue, no gender cue |
| **14** | Ein großer Mund [a big mouth] | Hund [dog] | no semantic cue, no gender cue |
| **15** | Ein lieber Junge [a nice boy] | Löwe [lion] | no semantic cue, no gender cue |
| **16** | Ein wilder Löwe [a wild lion] | Junge [boy] | semantic cue, no gender cue |
| **17** | Eine bunte Möwe [a colourful seagull] | Seife [piece of soap] | no semantic cue, no gender cue |
| **18** | Eine alte Robbe [an old seal] | Möwe [seagull] | no semantic cue, no gender cue |
| **19** | Eine gute Möwe [a good seagull] | Robbe [seal] | no semantic cue, no gender cue |
| **20** | Eine zarte Seife [a frail piece of soap] | Möwe [seagull] | semantic cue, no gender cue |
| **21** | Eine schöne Robbe [a beautiful seal] | Seife [piece of soap] | no semantic cue, no gender cue |
| **22** | Ein wilder Tiger [a wild tiger] | Käfer [bug] | semantic cue, no gender cue |
| **23** | Ein kleiner Käfer [a small bug] | Tiger [tiger] | semantic cue, no gender cue |
| **24** | Ein großer Affe [a big monkey] | Rabe [raven] | semantic cue, no gender cue |
| **25** | Ein alter Rabe [an old raven] | Affe [monkey] | no semantic cue, no gender cue |
| **26** | Ein schöner Apfel [a beautiful apple] | Löffel [spoon] | no semantic cue, no gender cue |
| **27** | Ein neuer Löffel [a new spoon] | Apfel [apple] | semantic cue, no gender cue |
| **28** | Eine bunte Kerze [a colourful candle] | Katze [cat] | no semantic cue, no gender cue |
| **29** | Ein feiner Löffel [a fine spoon] | Esel [donkey] | no semantic cue, no gender cue |
| **30** | Ein lieber Esel [a nice donkey] | Löffel [spoon] | semantic cue, no gender cue |
| **31** | Eine gute Butter [a good piece of butter] | Koffer [suitcase] | semantic cue, gender cue |
| **32** | Ein großer Koffer [a big suitcase] | Butter [piece of butter] | semantic cue, gender cue |
| **33** | Eine zarte Butter [a malleable piece of butter] | Teller [plate] | semantic cue, gender cue |
| **34** | Ein kleiner Teller [a small plate] | Butter [piece of butter] | semantic cue, gender cue |
| **35** | Eine neue Feder [a new feather] | Koffer [suitcase] | no semantic cue, gender cue |
| **36** | Ein alter Koffer [an old suitcase] | Feder [feather] | semantic cue, gender cue |
| **37** | Eine wilde Feder [a wild feather] | Teller [plate] | semantic cue, gender cue |
| **38** | Ein bunter Teller [a colourful plate] | Feder [feather] | no semantic cue, gender cue |
| **39** | Ein braver Hase [a well-behaved hare] | Tasse [cup] | semantic cue, gender cue |
| **40** | Eine schöne Tasse [a beautiful cup] | Hase [hare] | no semantic cue, gender cue |
| **41** | Ein lieber Junge [a nice boy] | Tasse [cup] | semantic cue, gender cue |
| **42** | Eine neue Tasse [a new cup] | Junge [boy] | semantic cue, gender cue |
| **43** | Eine brave Möwe [a well-behaved seagull] | Rabe [raven] | no semantic cue, gender cue |
| **44** | Ein lieber Rabe [a nice raven] | Möwe [seagull] | no semantic cue, gender cue |
| **45** | Ein alter Rabe [an old raven] | Robbe [seal] | no semantic cue, gender cue |
| **46** | Eine kleine Robbe [a small seal] | Rabe [raven] | no semantic cue, gender cue |
| **47** | Ein großer Junge [a small boy] | Katze [cat] | no semantic cue, gender cue |
| **48** | Eine bunte Katze [a colourful cat] | Junge [boy] | semantic cue, gender cue |
| **49** | Eine feine Katze [a fine cat] | Löwe [lion] | no semantic cue, gender cue |
| **50** | Ein schöner Löwe [a beautiful lion] | Katze [cat] | no semantic cue, gender cue |
| **51** | Eine zarte Seife [a frail piece of soap] | Käse [piece of cheese] | no semantic cue, gender cue |
| **52** | Ein guter Käse [a good piece of cheese] | Seife [piece of soap] | no semantic cue, gender cue |
| **53** | Ein alter Käse [an old piece of cheese] | Möwe [seagull] | no semantic cue, gender cue |
| **54** | Eine brave Möwe [a well-behaved seagull] | Käse [piece of cheese] | semantic cue, gender cue |
| **55** | Ein großer Affe [a big monkey] | Robbe [seal] | no semantic cue, gender cue |
| **56** | Eine kleine Robbe [a small seal] | Affe [monkey] | no semantic cue, gender cue |
| **57** | Ein lieber Igel [a nice hedgehog] | Nadel [needle] | semantic cue, gender cue |
| **58** | Eine feine Nadel [a fine needle] | Igel [hedgehog] | no semantic cue, gender cue |
| **59** | Eine neue Ampel [a new set of traffic lights] | Igel [hedgehog] | no semantic cue, gender cue |
| **60** | Ein wilder Igel [a wild hedgehog] | Ampel [set of traffic lights] | semantic cue, gender cue |
