# Supplementary material for: Online-Processing of Grammatical Gender in Noun-Phrase Decoding: An Eye-Tracking Study With Monolingual German 3rd and 4th Graders
Source: Front Psychol. 2019 Nov 15;10:2586. doi: 10.3389/fpsyg.2019.02586 (PMC6873886; doi:10.3389/fpsyg.2019.02586)
Supplement: Supplementary file 2 [file Table_2.docx]

Table S2

*Frequencies of the language stimuli per condition.*

|  |  | **Semantic cue** | | **No semantic cue** | | **Total** |
| --- | --- | --- | --- | --- | --- | --- |
|  |  | **No gender cue** | **Gender cue** | **Gender cue** | **No gender cue** |  |
| **Target nouns** | Ampel  Nadel  Apfel  Muschel  Esel  Butter  Feder  Hase  Junge  Löwe  Hund  Mund  Möwe  Robbe  Seife  Tiger  Käfer  Affe  Rabe  Löffel  Kerze  Koffer  Teller  Tasse  Katze  Käse  Igel | 0  0  1  1  2  0  0  1  0  2  1  0  0  0  1  1  1  1  0  1  0  0  0  0  0  0  0 | 0  0  0  0  0  2  1  1  1  0  0  0  1  0  0  0  0  0  0  0  0  2  1  1  1  0  2 | 1  1  0  0  0  0  1  0  1  1  0  0  1  2  1  0  0  1  2  0  0  0  1  1  1  2  0 | 2  1  1  0  0  1  1  1  2  0  0  1  2  2  0  0  0  0  1  1  1  0  0  0  0  0  0 | 3  2  2  1  2  3  3  3  4  3  1  1  4  4  2  1  1  2  3  2  1  2  2  2  2  2  2 |
| **Disctractor nouns** | Ampel  Nadel  Apfel  Muschel  Esel  Butter  Feder  Hase  Junge  Löwe  Hund  Mund  Möwe  Robbe  Seife  Tiger  Käfer  Affe  Rabe  Löffel  Kerze  Koffer  Teller  Tasse  Katze  Käse  Igel | 1  0  2  0  1  0  0  1  1  1  0  1  1  0  0  1  1  0  1  1  0  0  0  0  0  0  0 | 1  1  0  0  0  2  1  0  2  0  0  0  0  0  0  0  0  0  0  0  0  1  2  2  0  1  0 | 0  0  0  0  0  0  1  1  0  1  0  0  2  2  1  0  0  1  2  0  0  1  0  0  2  1  2 | 1  1  0  1  1  1  1  1  1  1  1  0  1  1  2  0  0  1  0  1  0  0  0  0  1  0  0 | 3  2  2  1  2  3  3  3  4  3  1  1  4  3  3  1  1  2  3  2  0  2  2  2  3  2  2 |
| **Adjectives** | bunt  klein  brav  lieb  alt  wild  zart  groß  neu  gut  schön  fein | 1  2  2  2  1  2  1  1  1  0  0  0 | 1  1  2  2  1  2  1  1  1  1  0  0 | 1  2  1  1  2  0  1  2  2  1  2  2 | 2  0  0  1  2  1  1  2  1  2  3  2 | 5  5  5  6  6  5  4  6  5  4  5  4 |
| **Articles** | Ein  Eine | 11  2 | 7  6 | 8  9 | 7  10 | 33  27 |
| **Number of items** |  | 13 | 13 | 17 | 17 | 60 |
